# Lateral ventricular cystic meningioma: 2 rare case reports

**DOI:** 10.3892/etm.2014.1550

**Published:** 2014-02-17

**Authors:** LI-HUA QIU, SU LUI, LING ZOU, QIANG YUE, QI-YONG GONG

**Affiliations:** 1 Huaxi MR Research Center (HMRRC), Department of Radiology, West China Hospital of Sichuan University, Chengdu, Sichuan 610041, P.R. China; 2 Department of Radiology, The Second People’s Hospital of Yibin, Yibin, Sichuan 644000, P.R. China

**Keywords:** cystic meningioma, intraventricular meningioma, magnetic resonance imaging, diagnosis

## Abstract

Cystic meningioma is an uncommon meningioma variant that is often difficult to distinguish from other intra-axial tumors, including necrotic gliomas. Cystic meningiomas located in the ventricle are particularly rare and may be misdiagnosed with other brain tumors, including ependymoma, choroid plexus papilloma and neurocytoma, due to its location. The present study discusses two cases of lateral ventricular meningiomas, which exhibited intratumoral or peritumoral cystic changes on magnetic resonance imaging scans. The two patients underwent surgical treatment and histological examination confirmed one case of metaplastic meningioma and the other case of psammomatous meningioma. The two patients were middle-aged females and had been misdiagnosed prior to surgery. Although this clinical entity is rare, the diagnosis of meningioma should be considered, particularly in middle-aged female patients.

## Introduction

Intraventricular meningiomas are rare tumors that account for 0.5–3% of all intracranial meningiomas (1), however, they remain one of the most challenging problems in neurosurgery. Among meningiomas of the central nervous system (CNS), cystic meningiomas are a distinct histological variant of meningiomas accounting for ~1.6% of all CNS meningiomas. Thus, cystic meningiomas located in the ventricle are particularly rare and may be misdiagnosed along with other brain tumors, including ependymoma, choroid plexus papilloma and neurocytoma. The present study discusses two cases of lateral ventricular meningiomas that exhibited intratumoral or peritumoral cystic changes with magnetic resonance imaging (MRI). The two cases were misdiagnosed prior to surgery. Written informed patient consent was obtained from the 2 patients. The aim of the present study was to provide additional data for this rare meningioma variant.

## Case reports

### Case 1

#### Patient history

A 48-year-old female presented with a chronic headache and vertigo that had persisted over a period of 2 months, but had progressively worsened in the last 10 days. Neurological examination, laboratory tests and other systemic evaluations (i.e., surgical and medical examination of the whole body) revealed no abnormalities, with the exception of hypertension.

#### Examination

**Figure 1:**
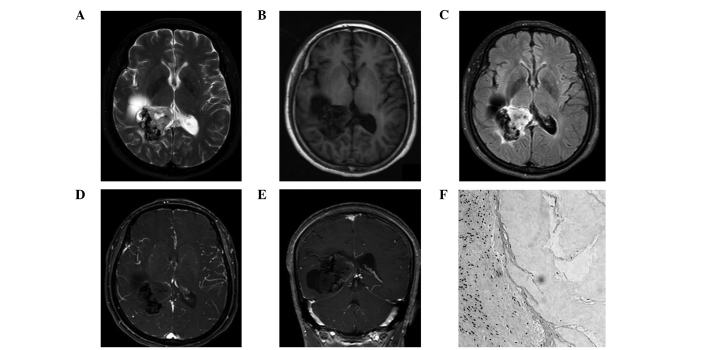
MRI scans revealed a 4.5×5.0×4.0 cm heterogeneously irregular mass in the trigone of the right lateral ventricle with slight peritumoral edema. (A–C) Noncontrast-enhanced MRI scans revealed that the solid portion of the tumor was hyperintense on T2WIs and T2-fluid attenuated inversion recovery images and hypointense on T1WIs. The cystic portion of the central tumor was hypointense on T1WIs and hyperintense on T2WIs. (D and E) Contrast-enhanced T1WIs revealed mild enhancement of the tumoral parenchyma following the injection of a contrast medium. Patchy calcification, which was revealed as hypointense in every sequence, was exhibited in the lateral marginal section of the tumor and scattered punctate calcification was also located within the solid area. (F) Microscopic examination of the resected tumor revealed that the left side was meningothelial cells and the right side was osseous tissue (hematoxylin and eosin staining; magnification, ×200). MRI, magnetic resonance imaging; WIs, weighted images.

MRI scans revealed a 4.5×5.0×4.0 cm heterogeneously irregular mass located in the trigone of the right lateral ventricle. An intratumoral cystic change was also observed. The tumoral parenchyma was hyperintense on T2-weighted images (WIs) and fluid attenuated inversion-recovery images, while appearing iso- to hypointense on T1WIs (Fig. 1A–E). Patchy calcification, which appeared hypointense in every sequence, was also observed in the marginal area of the tumor. Contrast enhanced MRI revealed mild enhancement in the tumoral parenchyma. The inferior and posterior horn of the right lateral ventricle revealed moderate obstructive hydrocephalus. Based on previous manifestations, the tumor was diagnosed as an ependyma or a choroid plexus papilloma prior to surgery.

#### Treatment

Surgery via temporal-occipital craniotomy revealed that the tumor was an ivory-colored hard mass which was well-circumscribed in the right trigone of the lateral ventricle. Evident calcification was observed without a visible blood supply. Pathological examination confirmed the mass to be a metaplastic meningioma [World Health Organization (WHO) grade I; Fig. 1F].

### Case 2

#### Patient history

A 43-year-old female presented with a persistent headache and blurred vision which has lasted for longer than one month. Neurological examination revealed papilla edematous and increased intracranial pressure.

#### Examination

**Figure 2:**
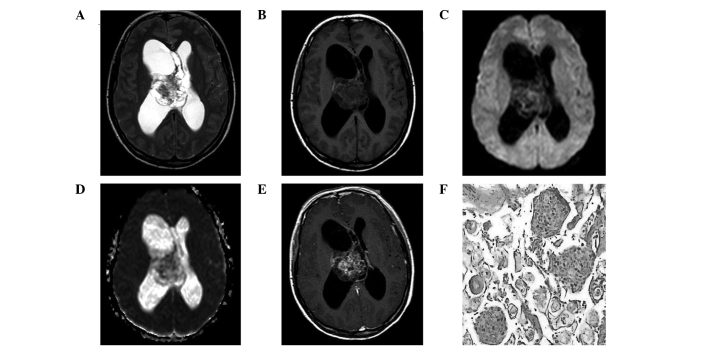
MRI scans revealed a heterogeneously cystic-solid mass with a size of 4.8×5.0×5.5 cm in the right lateral ventricle without peritumoral edema. (A–D) Noncontrast-enhanced MRI scans revealed that the solid area of the tumor was isointense on T1WIs, diffusion WIs and on an apparent diffusion coefficient map, and iso- to hypointense on T2WIs. (E) The tumoral parenchyma and septum demonstrated evident enhancement following the injection of gadolinium contrast media. (F) Pathological examination revealed excessive psammoma body formation (hematoxylin and eosin staining; magnification, ×400). MRI, magnetic resonance imaging; WIs, weighted images.

MRI scans revealed a heterogeneously cystic-solid mass of 4.8×5.0×5.5 cm in the right lateral ventricle. The solid area of the tumor exhibited an iso- to hypointense signal on T2WIs and an isointense signal on T1WIs and diffusion weighted imaging. On an apparent diffusion coefficient map, the parenchyma of the tumor exhibited an isointense signal. Overt enhancement was observed on T1-weighted post-gadolinium contrast images in the solid area instead of the cystic component (Fig. 2A–E). The tumor was diagnosed as an ependymoma or a neurocytoma prior to surgery.

#### Treatment

Surgery via a right temporoparietal craniotomy revealed that the tumor was a well-defined, yellowish in color, intact and encapsulated mass that adhered slightly to the walls of the lateral ventricle and the septum pellucidum. The marginal area of the tumor was cystic and the root originated from the choroid plexus. Pathological examination confirmed the mass to be a psammomatous meningioma (WHO grade I; Fig. 2F).

## Discussion

Meningiomas are the most common extra-axial neoplasm with the general characteristic imaging appearance of isointense or hypointense on T1WIs and isointense or hyperintense on T2WIs, exhibiting marked homogeneous contrast enhancement. Lateral ventricular and cystic meningiomas are rare meningioma variants (1). To the best of our knowledge, cystic changes in intraventricular meningiomas have not previously been reported in adult patients.

Cystic meningioma, used to describe meningiomas with intratumoral or peritumoral cysts, are uncommon and account for 2–4% of all intracranial meningiomas (2). Rengachary *et al* classified cystic meningiomas as intratumoral or extratumoral (3). Pathophysiological mechanisms of peritumoral cysts may be caused by loculated widened subarachnoid space, edema of the surrounding brain, demyelination or hemorrhaging near the tumor, while intratumoral cysts are the outcome of cystic degeneration, ischemic necrosis or hemorrhaging within the tumor. Cystic meningioma may often be misdiagnosed as other neoplasms, including gliomas or metastatic tumors, on preoperative MRI (4). However, due to the specific lesion location (the lateral ventricle), the tumors in the present study were misdiagnosed as ependymomas, choroid plexus papillomas or neurocytomas, which are the most common tumor types to appear with cysts and calcifications in the ventricular system.

Although differential diagnosis is difficult to separate cystic meningiomas from common tumors in the ventricular system, specific signs may aid differentiation. The majority of supratentorial ependymomas (70%) arise within the brain parenchyma of the cerebral hemispheres rather than from the ependymal cells lining the ventricular surfaces (5). In addition, neurocytomas tend to occur in younger patients and are located at the foramen of Monro and the roof of the lateral ventricle. Intraventricular choroid plexus papillomas are more common in childhood with occasional calcification of punctate foci in lesions. However, meningiomas are most common in adult females (6).

Notably, the current two cases had histologically different subtypes, which may be associated with their various MRI manifestations. Case 1 is a metaplastic meningioma, characterized by mesenchymal elements, including osseous, cartilaginous and myxoid tissue, which may lead to calcification or ossification and cystic formation as exhibited in the MRI scans. A previous case report (7) regarding a metaplastic meningioma in a child also revealed similar MRI features; for example, the meningioma was located in the lateral ventricle and cystic changes were observed within the tumor. By contrast, case 2 is of a psammomatous meningioma, which is mainly composed of hyalin and calcifies to form the characteristic concentric calcifications known as psammoma bodies, thus, is shown as a densely calcified mass on MRI and CT scans. The cystic-solid feature of psammomatous meningioma has not been reported previously. Thus, we hypothesized that the cystic change in this case may be associated with the penetration of cerebrospinal fluid around the tumor, which leads to the multiple cystic changes within the tumor. Metaplastic and psammomatous meningiomas are classified as WHO grade 1 with a low risk of recurrence and aggressive growth. Total tumor resectioning is important to reduce the risk of recurrence. The two patients were completely recovered without positive neurological symptoms and signs of recurrence at one year follow-up.

In conclusion, to the best of our knowledge, this is the first case report of cystic meningioma localized in the lateral ventricle in adult females. Although cystic meningioma located in the lateral ventricle is rare, this clinical entity should be included in the differential diagnosis of neoplasms in the lateral ventricle, particularly in adult female patients.
